# Rituximab treatment for refractory nephrotic syndrome in adults: a multicenter retrospective study

**DOI:** 10.1080/0886022X.2023.2237124

**Published:** 2023-07-24

**Authors:** Xiaoyan Ma, Lu Fang, Lili Sheng, Xun Zhou, Shoujun Bai, Xiujuan Zang, Yakun Wang, Mengke Li, Zexin Lv, Qin Zhong, Xinyu Yang, Yishu Wang, Yan Hu, Danying Yan, Yingfeng Shi, Hui Chen, Jinqing Li, Min Tao, Shougang Zhuang, Yi Wang, Na Liu

**Affiliations:** a Department of Nephrology, Shanghai East Hospital, Tongji University School of Medicine, Shanghai, P.R. China; b Department of Nephrology, Qingpu Branch of Zhongshan Hospital Affiliated to Fudan University, Shanghai, P.R. China; c Department of Nephrology, Shanghai Songjiang District Central Hospital, Shanghai, P.R. China; d Department of Medicine, Rhode Island Hospital and Alpert Medical School, Brown University, Providence, RI, USA

**Keywords:** Refractory nephrotic syndrome, rituximab, complete remission, partial remission, safety evaluation

## Abstract

**Background:**

The treatment of refractory nephrotic syndrome (RNS) is full of challenges and the role of rituximab (RTX) is not well-established, thus this study aims to demonstrate the role of RTX in RNS.

**Methods:**

This was a multicenter retrospective study of all adult patients receiving RTX for RNS. Patients enrolled were divided into two groups according to pathological pattern: 20 patients as a group of podocytopathy (including minimal change disease [MCD] and focal and segmental glomerulosclerosis [FSGS]), and 26 patients as membranous nephropathy (MN) group. The remission rate, relapse rate, adverse effects, and predictors of remission were analyzed.

**Results:**

A total of 75 patients received RTX for RNS and 48 were available for analysis after exclusion criteria. No significant difference in the remission rate at 6 or 12 months was observed between the MCD/FSGS and MN cases (*p* > 0.05). The median duration of the first complete remission (CR) was 1 month in the podocytopathy group and 12.5 months in the MN group. Three relapses were associated with infection as the ultimate outcome, and 6 out of 48 remained refractory representing a response rate of 87.5% in RNS. Clinical predictors of cumulative CR were estimated glomerular filtration rate (eGFR) <60 mL/min/1.73 m^2^ and mean arterial pressure (MAP) ≤103 mmHg at the beginning of therapy in patients with MN. No serious adverse effects were reported.

**Conclusions:**

RTX appears to be effective in RNS across various clinical and pathological subtypes, exhibiting a low relapse rate and minimal significant side effects in the majority of patients.

## 1. Introduction

Refractory nephrotic syndrome (RNS) is defined as patients with nephrotic syndrome who are steroid-resistant or steroid-dependent, immunosuppression-resistant, and frequently relapse [1–3]. In Asia, approximately 5 − 14% of RNS patients progress to end-stage renal disease (ESRD) within a span of 10–15 years [4]. Given the ineffective treatments, intricated patient profiles, and high relapse rates, there is an urgent demand for innovative therapeutic approaches to address these challenges.

The pathogenesis of idiopathic nephrotic syndrome (INS) has not been fully clarified, but accumulating evidence indicates that B cells play a crucial role in the development of glomerulopathy [1,5]. Rituximab (RTX), a monoclonal antibody binding specifically to the CD20 receptor on the cell membrane of precursor B cells and mature B cells, induces the depletion of B lymphocytes through apoptosis *via* antibody-dependent cellular cytotoxicity and complement-dependent cytotoxicity [6,7]. Recently, RTX has been applied in INS such as minimal change disease (MCD), focal and segmental glomerulosclerosis (FSGS), and membranous nephropathy (MN) [7–9]. According to the Kidney Disease Improving Global Outcomes (KDIGO) 2021 guideline, RTX is now the first-line treatment for patients with idiopathic MN with a response rate of 60–80% at 12 months [10]. Furthermore, emerging data support the effective role of RTX in the management of patients with steroid-dependent nephrotic syndrome *via* targeting B cells (as autoantibody-producing cells) [11,12]. However, the use of RTX to treat RNS remains controversial and challenging [12–15].

Considering the beneficial effects of RTX in the treatment of patients with INS, we designed this multicenter retrospective study to evaluate the efficacy and safety of RTX in RNS patients who were non-responsive to initial immunosuppression therapies or experienced recurrent relapse despite completing at least one full cycle of immunosuppression therapy. This study analyzed the remission rate, relapse rate, adverse effects, and predictors of remission in RNS patients, and aimed at demonstrating the role of RTX in the management of RNS.

## 2. Methods

### 2.1. Patients and ethics statement

**Figure 1.**
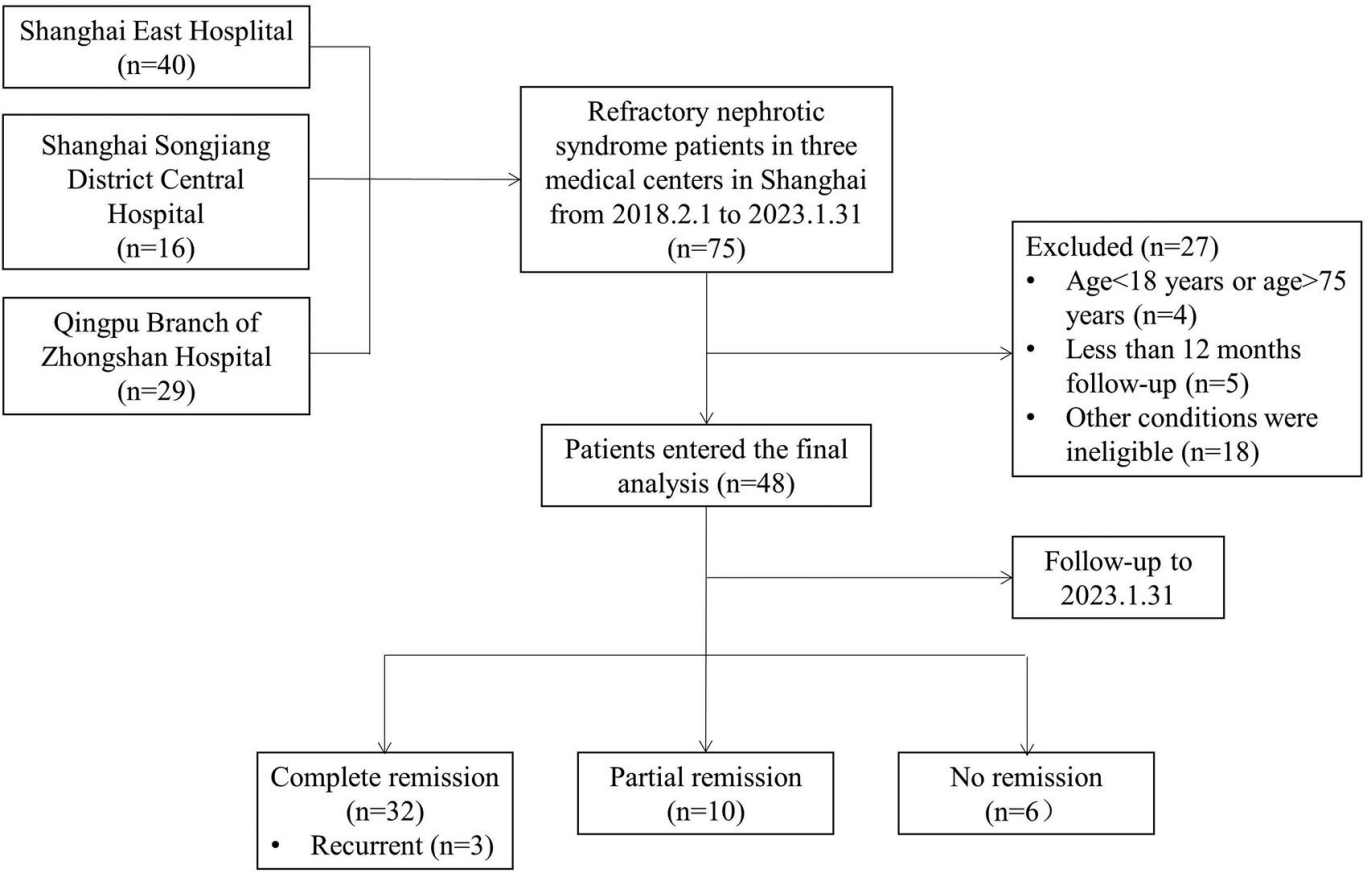
Study flow diagram.

The inclusion criteria of patients: adult patients who received RTX for RNS between 1 February 2018 and 31 January 2023 in Shanghai East Hospital, Shanghai Songjiang District Central Hospital, and Qingpu Branch of Zhongshan Hospital affiliated with Fudan University were all enrolled and were followed for a minimum period of 12 months. The average follow-up time was 3–6 months. Exclusion criteria: (1) with a secondary cause of the glomerular disease (autoimmune, infection, or malignant tumor, etc.); (2) with a serious infection, acute cardiovascular, or cerebrovascular disease; (3) with active hepatitis, cirrhosis, HBV or HCV infection or HIV infection; (4) currently or previously treated for tuberculosis; and (5) previously treated with RTX. Patients in the study received intravenous RTX at a dose of 375 mg/m^2^ once a week for four consecutive weeks, or intravenous RTX at a dose of 1 g every 2 weeks (a total of two times as a course of treatment). To minimize infusion reaction, patients received intravenous methylprednisolone (40 mg) and oral cetirizine (10 mg) before infusion. B cells were monitored before and after administration. Patients enrolled were divided into two groups according to a pathological pattern: 20 patients in group of podocytopathy (including MCD and FSGS), and 26 patients in MN group. MCD and FSGS were considered podocytopathies for their diffuse podocyte foot process effacement under electron microscopy. The pathologic type of the other two patients was membranoproliferative glomerulonephritis. The remission rate, relapse rate, adverse effects, and predictors of remission were analyzed. The study flow diagram was shown in Figure 1. The study protocol was approved by the Human Research Ethics Committee of Shanghai East Hospital Affiliated with Tongji University School of Medicine (ChiCTR2200055680).

### 2.2. Definitions and indications of therapy

RNS was defined according to criteria as follows: (1) Frequently relapsing: relapse ≥2 times in 6 months or ≥4 times in 12 months; (2) steroid dependent: relapse occurred within 2 weeks after steroid therapy; (3) MN or mesangial proliferative glomerular nephritis (MPGN) did not response to one round of immunosuppression; (4) steroid intolerant; and (5) steroid-resistant: MCD or FSGS did not respond to steroid treatment for 12 weeks.

### 2.3. Follow-up

B cells were monitored before and after the administration of RTX. CD19^+^-B-cell depletion was defined as less than 5 cells/µL. The dose of RTX was adjusted according to the condition of the patients by the treating physicians. We measured IgG levels before and after giving RTX infusion. Since RTX infusion may decrease the IgG level [16] and low IgG level (<6 g/L) is associated with serious infections [17], we will give supplementary intravenous immunoglobulin to restore IgG to 6 g/L while IgG is less than 6 g/L.

We searched the database of each medical center to collect the data of the enrolled patients before RTX administration and at four-time points (3, 6, 9, and 12 months) after the last dose of RTX. After 6 months, at least one dose of RTX (375 mg/m^2^) therapy may be repeated according to CD19^+^-B-cell count. Follow-up ended on 31 January 2023 or when patients received other immunosuppressant or received dialysis or died. The remission, relapse, and treatment inefficiency of each patient were recorded; the remission rate and relapse rate were counted; the effects of RTX treatment between MCD/FSGS and MN groups were compared.

### 2.4. Responses and outcomes

The primary endpoint was the percentage of patients with complete or partial remission of nephrotic syndrome after a 12 month follow-up period. Secondary endpoints included the relapse rate, doubling of the baseline serum creatinine concentration and adverse effects at the end of the observation.

To evaluate therapeutic responses, remission was defined according to 2021 KDIGO guideline [17] as (1) complete remission (CR) was defined as a reduction in 24-h proteinuria to ≤0.3 g with normal kidney function; (2) partial remission (PR) as a reduction in proteinuria of more than 50% from baseline with a value of 24-h proteinuria less than 3.5 g and normal kidney function; (3) no response was defined as a proteinuria reduction of less than 50% from baseline values or not meeting the criteria for CR or PR. Relapses were defined as a recurrence of proteinuria >3.5 g/24-h or >50% increase from the lowest value achieved during PR or CR.

### 2.5. Statistical analysis

Normally distributed data were presented as mean ± SD, while non-normally distributed data were presented as median with interquartile range (IQR). Differences in qualitative data were compared using a ‘Chi-squared’ test or Fisher’s exact test. The Kaplan–Meier method was used to plot the probability of CR, and the log-rank test was used to compare curves. Survival time was determined from the beginning of the RTX treatment until the first event of interest. Patients who had not achieved the event were considered censored at the time of the last visit. Univariate and multivariate Cox regression analyses were performed to confirm potential risk or protection factors of treatment responses. Variables that were statistically significant with a *p* value <0.15 in univariate Cox models were further assessed with multivariate Cox models using an enter method. The results were presented as adjusted hazard ratios (AHRs) with 95% confidence intervals (CIs). All probabilities were two-tailed and the level of significance was set at 0.05. Statistical analysis was performed using SPSS version 19.0 (SPSS Inc., Chicago, IL).

## 3. Results

### 3.1. Patients

A total of 75 patients were identified with RTX therapy for RNS. After a median follow-up period of 18.5 (12.5, 29.25) months, 48 patients entered the final analysis according to the inclusion and exclusion criteria (Figure 1). The baseline demographic and clinical characteristics were shown in Table 1. Of a total of 48 eligible patients with an average age of 47.88 ± 16.93 years old, 33 were males and 8 were smokers. Nine patients had diabetes, and 22 had hypertension. The average blood pressure was 135.08 ± 17.34/82.75 ± 10.74 mmHg. Median disease duration of 48 patients was 29.5 (IQR 1260) months. All the patients were with biopsy-proven INS, 15 (31.3%) with MCD, 5 (10.4%) with FSGS, 26 (54.2%) with MN, and 2 (4.2%) with MPGN. The immunosuppressive therapies of patients before RTX treatment were steroid only (12, 25%), cyclosporine or tacrolimus (24, 50%), cyclophosphamide (10, 20.8%), mycophenolate (1, 2.1%), and others (1, 2.1%). One patient with other immunosuppressive agents was a male with MCD who received steroid and leflunomide therapy. All patients had received at least one course of immunosuppression therapy, with a median of 2 (IQR 1–3) courses. The reasons of refractory for patients enrolled included steroid dependent (4, 8.3%), steroid-resistant (3, 6.3%), frequent relapsing (11, 22.9%), steroid intolerant (5, 10.4%), and resistance to immunosuppression (25, 52.1%).

**Table 1.** Baseline characteristics of study population before rituximab therapy.

Characteristic	Patients (*n* = 48)
Male sex, *n* (%)	33 (68.8)
Age, years	47.88 ± 16.93
Smoker, *n* (%)	8 (16.7)
Drinker, *n* (%)	6 (12.5)
BMI, kg/m^2^	24.85 ± 3.52
Systolic BP, mmHg	135.08 ± 17.34
Diastolic BP, mmHg	82.75 ± 10.74
Serum creatinine, µmol/L	94.65 ± 44.58
eGFR, mL/min/1.73 m^2^	84.65 ± 32.69
Proteinuria, g/24 h	6.77 ± 3.9
<4 g, *n* (%)	13 (27.0)
4–8 g, *n* (%)	19 (39.6)
>8 g, *n* (%)	26 (33.3)
Albumin, g/L	24.56 ± 6.55
Total cholesterol, mmol/L	6.09 (4.46,7.62)
Triglycerides, mmol/L	2.19 (1.50,3.54)
Receiving ACEI/ARB, *n* (%)	37 (77.1)
Receiving statin, *n* (%)	31 (64.6)
Disease duration, months	29.5 (12,60)
Reason of refractory	
Steroid dependent, *n* (%)	4 (8.3)
Steroid resistant, *n* (%)	3 (6.3)
Frequently relapsing, *n* (%)	11 (22.9)
Steroid intolerant, *n* (%)	5 (10.4)
Resistant to immunosuppression, *n* (%)	25 (52.1)
Immunosuppressive therapy before RTX	
Steroid only, *n* (%)	12 (25.0)
Cyclosporine or tacrolimus, *n* (%)	24 (50.0)
Cyclophosphamide, *n* (%)	10 (20.8)
Mycophenolate, *n* (%)	1 (2.1)
Others, *n* (%)	1 (2.1)
Prior immunosuppressive therapy	
Steroid only, *n* (%)	12 (25.0)
One round of immunosuppression, *n* (%)	10 (20.8)
Two rounds of immunosuppression, *n* (%)	13 (27.1)
Three rounds of immunosuppression, *n* (%)	13 (27.1)
Renal histologic pathology	
MCD, *n* (%)	15 (31.3)
FSGS, *n* (%)	5 (10.4)
MN, *n* (%)	26 (54.2)
MPGN, *n* (%)	2 (4.2)
Diabetes, *n* (%)	9 (16.7)
Hypertension, *n* (%)	22 (45.8)

ACEI: angiotensin-converting enzyme inhibitor; ARB: angiotensin receptor antagonist; eGFR: estimated glomerular filtration rate; FSGS: focal segmental glomerulosclerosis; MCD: minimal change disease; MN: membranous nephropathy; MPGN: membranoproliferative glomerulonephritis

Before RTX treatment, the average serum creatinine of the 48 patients was 94.65 ± 44.58 µmol/L, with an estimated glomerular filtration rate (eGFR) of 84.65 ± 32.69 mL/min/1.73 m^2^. The average proteinuria was 6.77 ± 3.9 g/24-h, with 26 of 48 patients (33.3%) having proteinuria exceeding 8 g/24-h. The serum albumin was 24.56 ± 6.55 g/dL. A total of 37 patients (77.1%) received angiotensin-converting enzyme inhibitor (ACEI)/angiotensin receptor antagonist (ARB) and 31 (64.6%) received statin. The laboratory parameters of patients after RTX therapy were shown in Supplementary Table 1.

### 3.2. Treatment response and outcomes

The outcome of these 48 patients after RTX therapy was shown in Figure 1. Out of the 48 patients, 32 (66.7%) achieved CR, 10 (20.9%) achieved PR and 6 (12.5%) patients had no remission (including one patient who progressed to ESRD requiring dialysis) until the end of the follow-up period.

**Figure 2.**
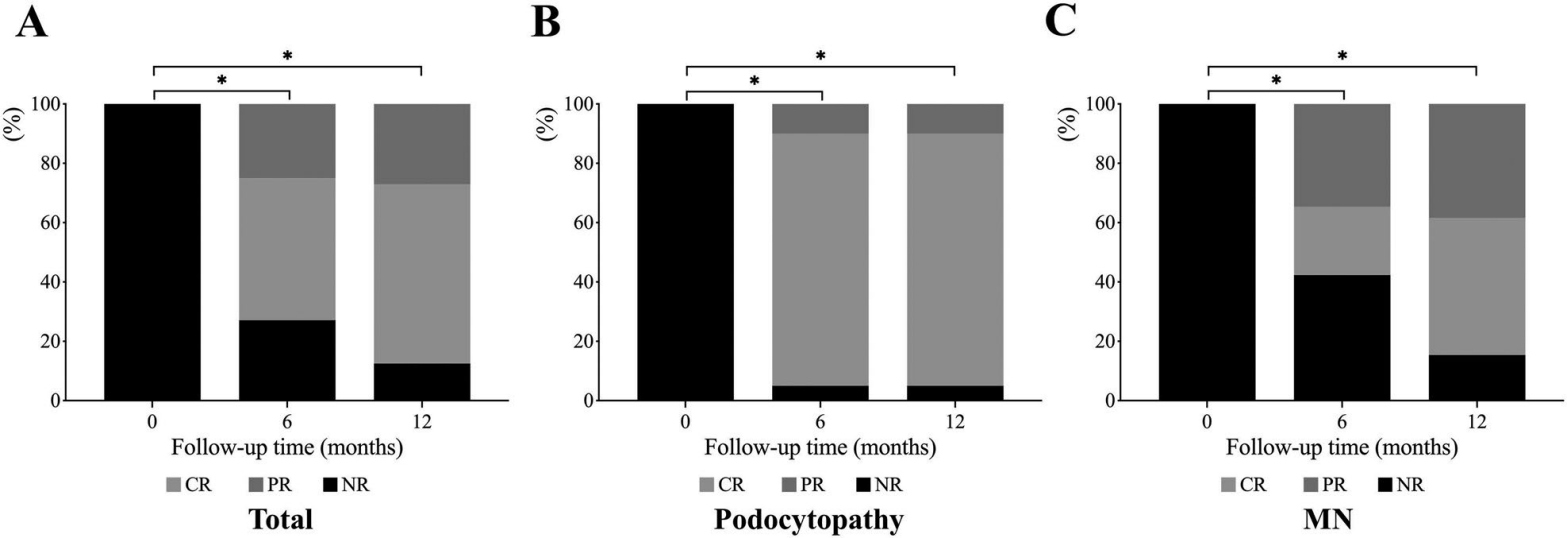
The efficacy outcome in the 12-month follow-up. Histogram showed the percentage of patients who reached PR, CR, or NR. (A) outcome of all patients. (B) outcome of patients with podocytopathy (MCD and FSGS). (C) outcome of patients with MN. *p < 0.05 complete remission rates compared to baseline. PR: partial remission; CR: complete remission; NR: no response; MCD: minimal change disease; FSGS: focal and segmental glomerulosclerosis; MN: membranous nephropathy.

Patients enrolled were divided into two groups according to their pathological patterns: 20 patients in a group of podocytopathy (including MCD and FSGS), and 26 patients in MN group. The 6 and 12 months outcomes of each group were shown in Figure 2. In podocytopathy group, 19/20 (95.0%) patients reached remission at 6 and 12 months (17 patients reached CR and 2 patients reached PR). While, in the MN group, 15/26 (57.7%) patients achieved remission at 6 months after RTX treatment, with 6/26 (23.1%) CR and 9/26 (34.6%) PR. And at 12 months, 22/26 (84.6%) patients of MN group reached remission with 12/26 (46.2%) achieving CR and 10/26 (38.5%) PR. The percentage of patients who reached remission between the two groups was of no difference at 6 and 12 months (*p* > 0.05). There were three relapses in 1 year, two patients in podocytopathy group (2/20, 10%) and one in MN group (1/26, 3.8%). The reasons for relapses were bacterial pneumonia, lymphangitis and viral pneumonia respectively. Patients who achieved CR or PR after RTX treatment without recurrence did not switch to other steroids and immunosuppressive regiments. In podocytopathy group, two relapsed patients received additional steroid treatment and finally achieved CR. However, three patients without CR (including two PR patients and one patient without remission) continued to receive RTX treatment by adding 1000 mg RTX for a half year but still failed to achieve CR. In MN group, after RTX treatment, one relapsed patient achieved CR after adding CTX, three patients without CR were changed to steroids and CTX therapy but still did not achieve CR, and eight patients without CR continued to receive RTX treatment and six patients finally achieved CR.

The outcomes of patients with different reasons for refractory in 12 months were described in Supplementary Table 2. The PR rate of patients with steroid resistance was 66.7% (none of these patients achieved CR during the observation period). The CR rates of patients with steroid-dependent, frequently relapsing, steroid intolerant and resistant to immunosuppression were 100.0%, 100.0%, 40.0%, and 36.0% at 12 months. None of the patients with steroid dependent or steroid intolerant relapsed after the treatment of RTX while the relapse rates of patients with frequently relapsing or resistant to immunosuppression were 18.2% and 4.0% until the end of the follow-up period.

### 3.3. Factors affect complete remission in MN patients

**Figure 3.**
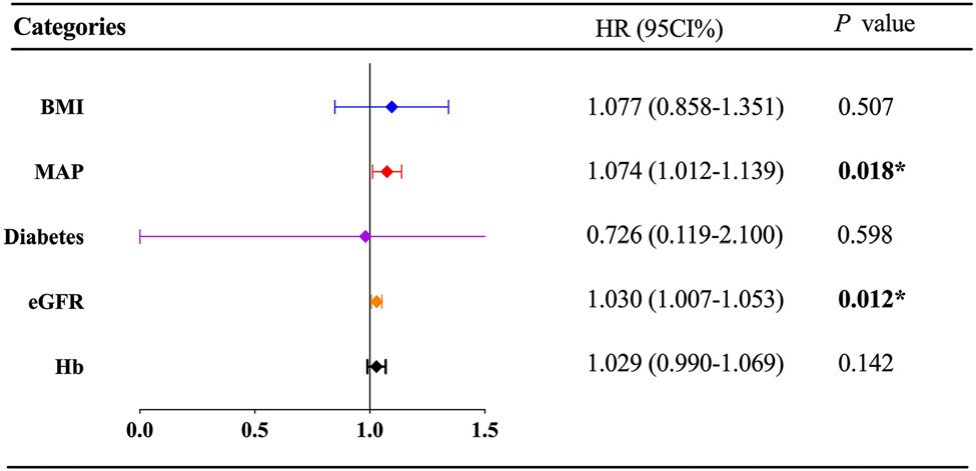
The independent Prognosis factors of complete remission in patients with MN receiving rituximab. The forest plot of Cox proportional hazard analysis showed the results of multivariate Cox proportional hazard analysis on the prognosis factors of complete remission in patients with MN receiving rituximab. BMI: body mass index; MAP: mean arterial pressure; eGFR: estimated glomerular filtration rate; Hb: hemoglobin. 95% CI: 95% confidence interval; HR: hazard ratio. *p* < 0.05 was considered to be statistically significant.

**Figure 4.**
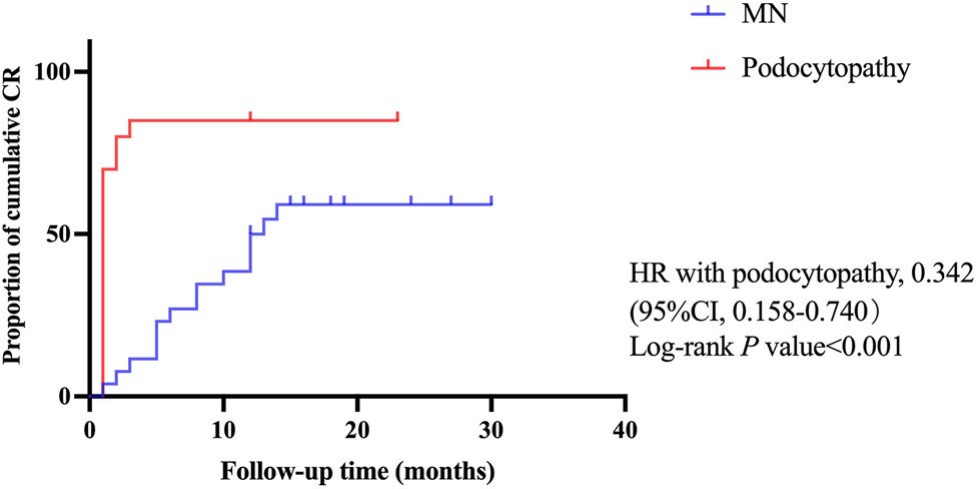
Kaplan–Meier curves for the complete remission after Rituximab in patients with MN and podocytopathy. Kaplan–Meier curves were shown for the proportion of cumulative complete remission during the follow-up period in patients receiving Rituximab with membranous nephropathy (MN) compared with the patients in podocytopathy group. 95% CI: 95% confidence interval; HR: hazard ratio. *p* < 0.05 was considered to be statistically significant.

According to KDIGO 2021 guideline [17], the risk classification of MN was divided into low risk (24-h proteinuria <4 g), medium risk (4 g ≤ 24-h proteinuria ≤8 g) and high risk (24-h proteinuria >8 g). We continue to use this as the standard, and the proportion of patients with proteinuria risk has been shown in Table 1. As the CR of MN took a long time, we conducted a Cox regression analysis to identify factors impacting its CR. The univariate Cox regression analysis showed eGFR level (HR 1.040, 95% CI 1.001–1.041, *p* = 0.04) was positively associated with CR rate (Table 2) while the different grades of proteinuria were not (*p* > 0.05). In the multivariate model (Figure 3), eGFR level was still an independent factor for CR (HR 1.030, 95% CI 1.007–1.053, *p* = 0.012); in addition, MAP was also an independent factor for CR (HR 1.074, 95% CI 1.012–1.139, *p* = 0.018). These results suggested that decreased eGFR and lower MAP were independent risk factors for cumulative CR. Further Kaplan–Meier analysis showed that patients with eGFR < 60 mL/min/1.73 m^2^ at baseline had a longer time to achieve CR and a lower cumulative CR rate than patients with eGFR ≥ 60 mL/min/1.73 m^2^ (*p* = 0.02, Supplementary Figure 1). The average blood pressure of MN patients was 144.23 ± 13.37/86.50 ± 9.30 mmHg, the average MAP was 105.74 ± 9.35 mmHg. Based on the average MAP, we selected 103 mmHg as a cutoff point. Kaplan–Meier analysis showed that patients with MAP ≤ 103 mmHg had a longer time to achieve CR and a lower cumulative CR rate than patients with MAP > 103 mmHg (*p* = 0.034, Supplementary Figure 2). In addition, Kaplan–Meier analysis (Figure 4) showed that the median first CR duration of patients in podocytopathy group was one month, while that of patients in MN group was 12.5 months. Patients in podocytopathy group tended to reach early remission (*p* < 0.001).

**Table 2.** Prognosis factors of complete remission in patients with MN receiving rituximab based on the results of univariate Cox proportional hazard analysis.

	HR	HR (95% CI)	*p* Value
Gender	1.364	(0.434–4.289)	0.596
Age (per year)	1.011	(0.975–1.049)	0.547
Duration (per month)	0.994	(0.979–1.009)	0.415
BMI (per 1 kg/m^2^)	1.192	(0.969–1.465)	0.096
Smoker	0.958	(0.271–3.444)	0.958
Drinker	1.391	(0.311–6.182)	0.664
MAP (per 1 mmHg)	1.059	(0.996–1.127)	0.067
Hypertension	2.307	(0.733–7.262)	0.153
Diabetes	0.288	(0.064–1.284)	0.103
eGFR (per 1 mL/min/1.73 m^2^)	1.040	(1.001–1.041)	0.040*
URBC (per 1/µL)	1.005	(0.997–1.014)	0.176
Hb (per 1 g/L)	1.031	(0.995–1.069)	0.086
Alb (per 1 g/L)	1.021	(0.955–1.091)	0.542
ALT (per 1 IU/L)	1.026	(0.990–1.063)	0.153
TC (per 1 mmol/L)	0.953	(0.760–1.194)	0.675
TG (per 1 mmol/L)	0.915	(0.683–1.224)	0.549
Upro (per 1 grade)	0.873	(0.441–1.729)	0.697
CRP (per 1 mg/L)	0.938	(0.806–1.090)	0.403

BMI: body mass index; MAP: mean arterial pressure; eGFR: estimated glomerular filtration rate; URBC: urine red blood cell; Hb: hemoglobin; Alb: albumin; ALT: alanine transaminase; Upro: urine protein; TG: triglyceride; TC: total cholesterol; CRP: c-reactive protein; 95% CI: 95% confidence interval; HR: hazard ratio

Proteinuria was divided into three grades, less than 4 g is grade 1, 4–8 g is grade 2, and greater than 8 g is grade 3.

*p* < 0.05 was considered statistically significant.

### 3.4. Adverse effects

RTX was well tolerated in almost all patients. With methylprednisolone and cetirizine administration before infusion, no severe adverse event was observed during infusion in all patients. In this study, no patients treated with RTX developed agranulocytosis, cytopenia, infusion reactions, hypertension, and gastrointestinal pain. One patient occurred nausea and vomiting, one patient suffered from carbuncle, four patients experienced slightly elevated ALT/AST, two patients experienced urinary tract infection, two patients developed pneumonia, five patients occurred rash, and one patient developed influenza-like symptoms as demonstrated in Table 3. All patients were treated appropriately.

**Table 3.** Adverse events of rituximab during follow-up.

Events	Patients (*n* = 48)
Hypertension	0
Gastrointestinal pain	0
Nausea or vomiting	1
Carbuncle	1
Abnormal ALT/AST	4
Urinary tract infection	2
Pneumonia	2
Rash or pruritis	5
Influenza-like symptoms	1
Headache or dizziness	0
Angioedema	0
Leucopenia or neutropenia	0

ALT: alanine aminotransferase; AST: aspartate aminotransferase

## 4. Discussion

In this multicenter retrospective study, the remission rate of all patients after RTX treatment was 87.5% at the end of the follow-up period. In podocytopathy group, 95.0% of patients reached remission. While in the MN group, 84.6% of patients reached remission at 12 months. The PR rate of patients with steroid-resistance was 66.7% (none of these patients achieved CR during the observation period). The CR rates of patients with steroid-dependent, frequently relapsing, steroid intolerant and resistant to immunosuppression were 100.0%, 100.0%, 40.0%, and 36.0% at 12 months. These findings suggested that RTX could significantly increase the response rate in RNS patients with different pathological or refractory reasons. In addition, the probability of CR in MN patients was associated with baseline eGFR level and MAP.

RTX has been used in various glomerulonephritis, including MCD, FSGS, MN as well as in MPGN. Guidelines have proposed RTX to be a priority choice to treat MN patients of moderate or high risk [18]. For MN patients, despite methodological differences, different treatment protocols and inconsistent criteria to define remission, overall remission rates of RTX at 12 months could approach 60–70% [6,19]. A meta-analysis involving 542 patients diagnosed as MN indicates that RTX could improve the total remission rate, achieving a higher rate of CR and reducing the level of PLA2R antibody [20]. However, the RI-CYCLO randomized trial demonstrated that there are no significant benefits or less harm associated with RTX therapy, compared with traditional immunosuppression therapy in the treatment of MN [21]. These studies focused on MN patients, and the efficacy of RTX in MN patients was controversial compared with traditional therapies. Recently, some researchers have indicated that RTX monotherapy may lead to satisfactory outcomes in a subset of patients with RNS [6,22,23]. As for podocyte diseases, in a study of MCD or FSGS patients with frequently relapsing, steroid-dependent or steroid-resistant, continuous B-cell depletion with RTX is beneficial to maintain remission [12]. Therefore, we conducted a small retrospective study to explore the efficacy of RTX in patients with RNS. In our research, we found overall remission rates in RNS and overall relapse rates of RNS were high across most pathological and clinical groups, except six patients with mostly MN. Low MAP and low eGFR were not conducive to RNS patients achieving remission while proteinuria did not affect the CR rate.

In our study, 6 (12.5%) patients resulted in no remission in the overall follow-up period, and one of them finally progressed to ESRD requiring kidney replacement therapy. Four out of six no-remission patients were biopsy-proven MN and the average disease duration of these patients was nearly 3 years, what’s more, these patients exhibited impaired kidney function at the beginning of the study. Further Kaplan–Meier analysis showed that patients with eGFR < 60 mL/min/1.73 m^2^ at baseline had a longer CR time and a lower cumulative CR rate than patients with eGFR ≥ 60 mL/min/1.73 m^2^. Low eGFR especially below 60 mL/min/1.73 m^2^ is a known predictor [24], indicating that RTX is not expected to be as effective, especially in MN. In addition, eGFR level may have an impact on the pharmacokinetics and tolerability of RTX [25]. For MN patients with kidney insufficiency, it is still controversial whether RTX can effectively reduce proteinuria, preserve kidney function, and delay or even reverse the progression of renal failure [6,26,27].

Our study also indicated that maintaining MAP and kidney perfusion may help RNS patients obtain CR. In the MN group, patients with MAP ≤ 103 mmHg had a longer CR time and a lower cumulative CR rate than patients with MAP > 103 mmHg. We noticed that patients in the MN group had higher baseline blood pressure values (144.23 ± 13.37/86.50 ± 9.30 mmHg) and a higher average MAP (105.74 ± 9.35 mmHg), which might be related to inadequate control of blood pressure prior to the onset of the disease. Patients with higher MAP at baseline with a better response to treatment might be associated with more benefit obtained from subsequent blood pressure control with ARB, ACEI or calcium channel blockers than those with lower blood pressure. Kidney function is regulated by the blood flow to and from the kidney system, which is determined by differences in intravascular pressure, and acute changes in MAP may lead to a decrease in GFR [28]. Maintaining macrovascular hydrostatic parameters such as MAP and central venous pressure is beneficial in preserving kidney perfusion and reducing the risk of acute kidney injury [29]. Compared with brain and heart, the minimum perfusion threshold related to organ function maintenance in kidney is higher [30]. It may be optimal to maintain the MAP slightly above the lower limit required to maintain kidney autoregulation [29], and low MAP (below 60–80 mmHg) may impair the automatic regulation of the kidney [31–33].

In our study, RTX appears to be effective in RNS across various clinical and pathological subtypes, exhibiting a low relapse rate and minimal significant side effects in the majority of patients. Indeed, the value of RTX is increasingly recognized for otherwise treatment-resistant renal vasculitis [34] or relatively novel, unusual considerations, such as checkpoint inhibitor-induced renal disease [35]. Moreover, RTX has shown a small incidence of adverse events and toxicity in many studies [36]. The major limitations of this study were the small sample size and the short duration of follow-up. Also, treatment protocols varied among patients with responses. Nevertheless, the results clearly showed the benefit of RTX treatment. A larger sample size and a stricter protocol are needed for future studies.

## 5. Conclusion

RTX was highly effective in various subgroups, except long-standing diseases with already low eGFR, and low MAP, especially with MN. In addition, our study is a retrospective study, and prospective studies based on this are warranted.

## Supplementary Material

Supplemental Material

Supplemental Material

Supplemental Material
