# Supplementary figures and images for: Rituximab treatment for refractory nephrotic syndrome in adults: a multicenter retrospective study
Source: Ren Fail. 2023 Jul 24;45(1):2237124. doi: 10.1080/0886022X.2023.2237124 (PMC10367573; doi:10.1080/0886022X.2023.2237124)

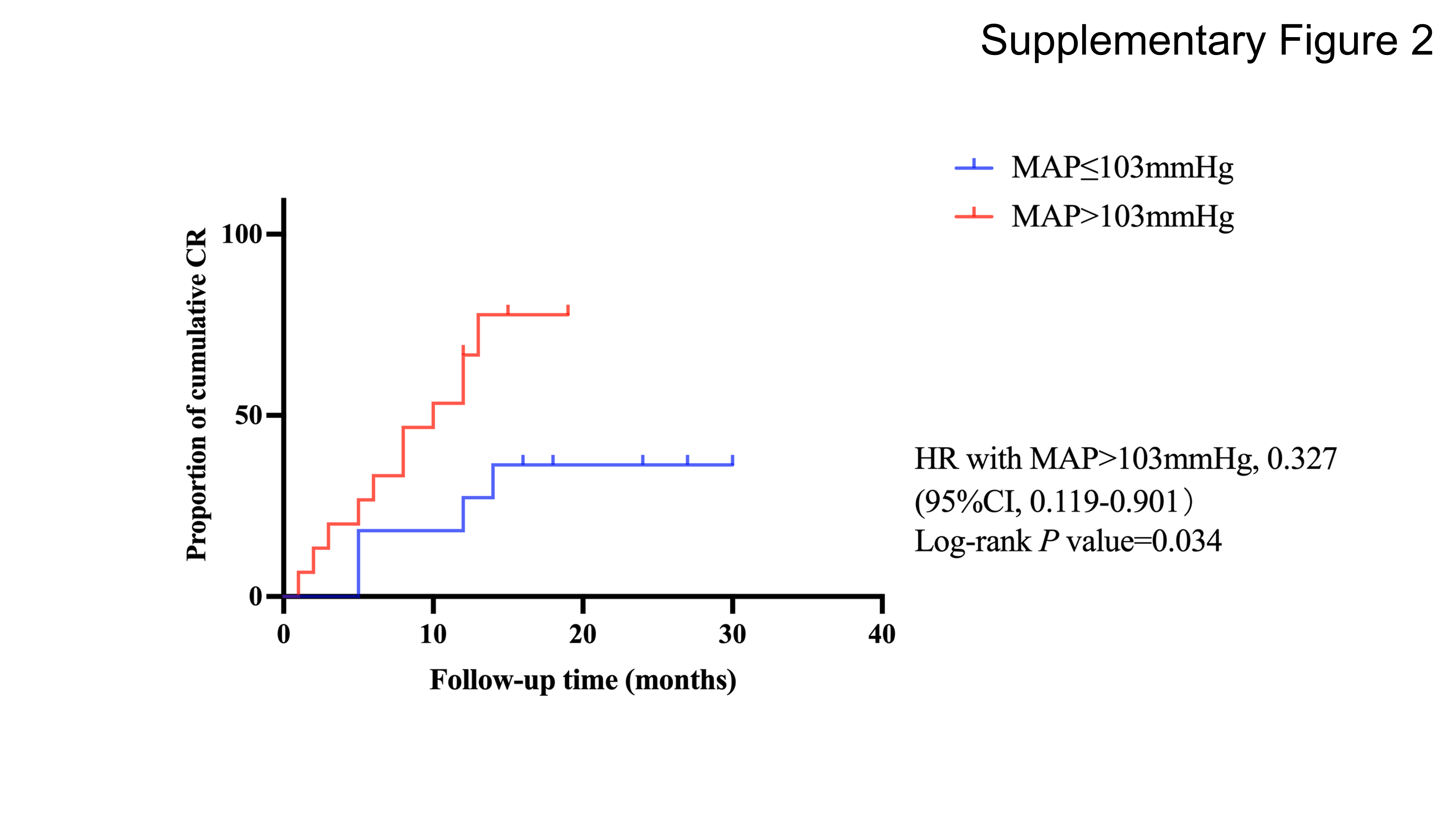

Supplement: Supplemental Material [file IRNF_A_2237124_SM7268.tif]

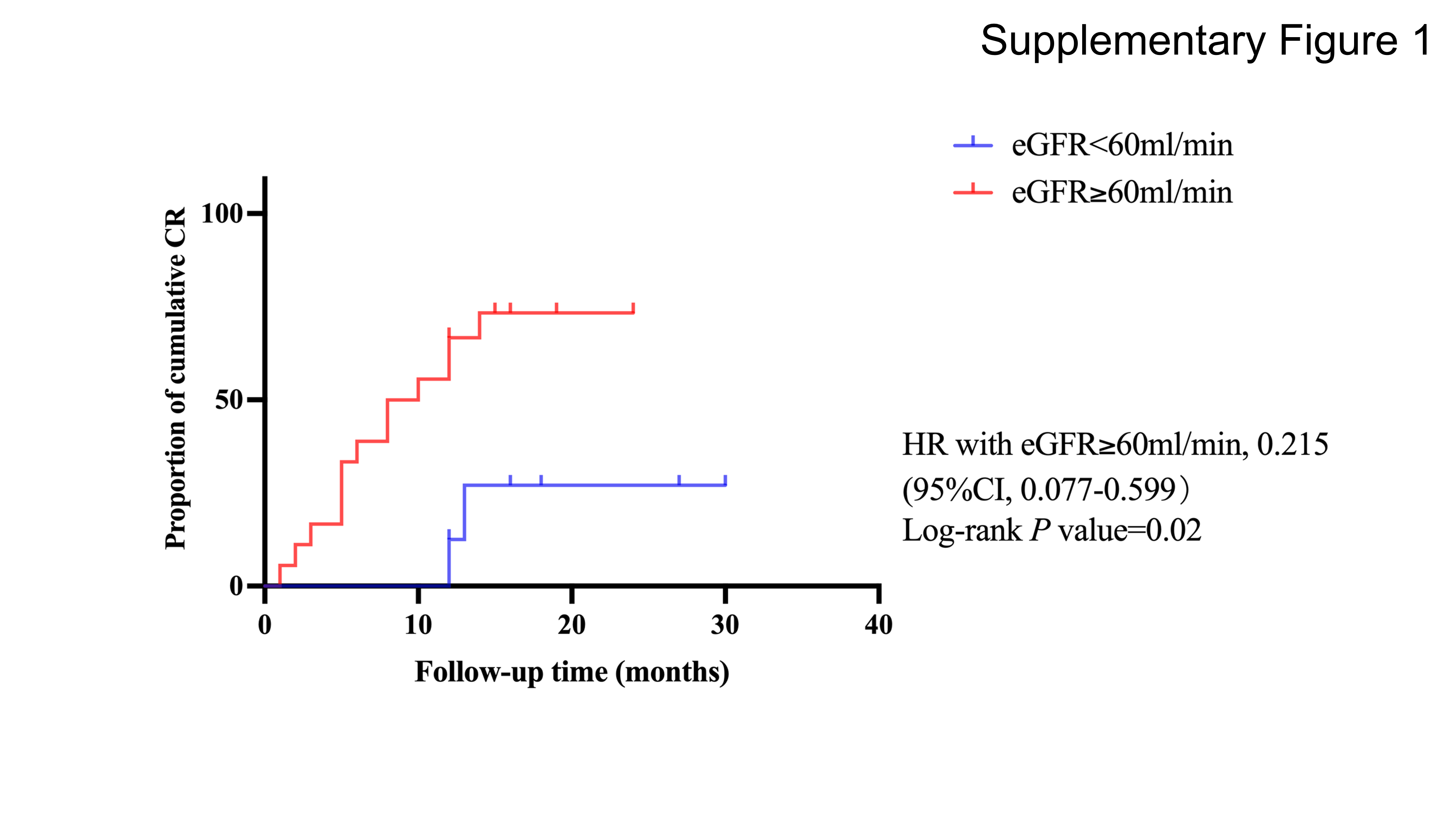

Supplement: Supplemental Material [file IRNF_A_2237124_SM7252.tif]
