# Supplementary material for: Rituximab treatment for refractory nephrotic syndrome in adults: a multicenter retrospective study
Source: Ren Fail. 2023 Jul 24;45(1):2237124. doi: 10.1080/0886022X.2023.2237124 (PMC10367573; doi:10.1080/0886022X.2023.2237124)
Supplement: Supplemental Material [file IRNF_A_2237124_SM7230.pdf]

Supplementary Table 1 Laboratory parameters before and after Rituximab

Laboratory parameters	Before Rituximab	After Rituximab	*P* value
Serum creatinine, μmol/L	94.65±44.58	102.86±100.19	0.528
eGFR, mL/min/1.73m^2	84.65±32.69	88.94±33.68	0.605
Proteinuria, g/24h	6.77±3.9	0.30 (0.14,1.86)	**0.000**
Albumin, g/L	24.56±6.55	41.21±6.61	**0.000**
Total cholesterol, mmol/L	6.09 (4.46,7.62)	4.67 (3.26,5.84)	**0.001**
Triglycerides, mmol/L	2.19 (1.50,3.54)	2.01 (1.05,3.82)	0.654
Hemoglobin, g/L	123.61±25.43	129.79±17.36	0.167
Leukocyte, (×10^5)	8.26±3.08	6.68±2.46	**0.007**
Lymphocyte, (×10^5)	2.48±4.05	2.26±1.59	0.739
Immunoglobulin G, g/L	5.92±2.55	8.26±2.06	**0.000**
Glycosylated hemoglobin	5.71±0.53	5.63±0.61	0.559

Note: P<0.05 is considered to be statistically significant.

Information Classification: General

Supplementary Table 2 Outcomes of patients with different reasons of refractory in 12 months

Outcome in 12 months	Steroid dependent (n=4)	Steroid resistant (n=3)	Frequently relapsing (n=11)	Steroid intolerant (n=5)	Resistant to immunosuppression (n=25)
CR, n (%)	4 (100.0)	0 (0)	11 (100.0)	2 (40.0)	9 (36.0)
PR, n (%)	0 (0)	2 (66.7)	0 (0)	2 (40.0)	11 (44.0)
NR, n (%)	0 (0)	1 (33.3)	0 (0)	1 (20.0)	5 (20.0)
Relapse, n (%)	0 (0)	0 (0)	2 (18.2)	0 (0)	1(4.0)

Abbreviations: CR, complete remission; PR, partial remission; NR, no remission.

Information Classification: General
